# Supplementary material for: Roles of Progranulin and FRamides in Neural Versus Non-Neural Tissues on Dietary Restriction-Related Longevity and Proteostasis in C. elegans
Source: J Clin Med Sci. Author manuscript; Available in PMC 2024 Sep 25. (PMC11423770)
Supplement: Supplementary [file NIHMS2007370-supplement-Supplementary.pdf]

ISSN: 2593-9947
Journal of Clinical and Medical Sciences

OPEN ACCESS Freely available online

Research Article

Roles of Progranulin and FRamides in Neural *Versus* Non-Neural Tissues on Dietary Restriction-Related Longevity and Proteostasis in *C. elegans*

Dilawar Ahmad Mir*, Matthew Cox, Jordan Horrocks, Zhengxin Ma, Aric Rogers

Kathryn W. Davis Center for Regenerative Biology and Aging, Mount Desert Island Biological Laboratory, Maine, United States of America

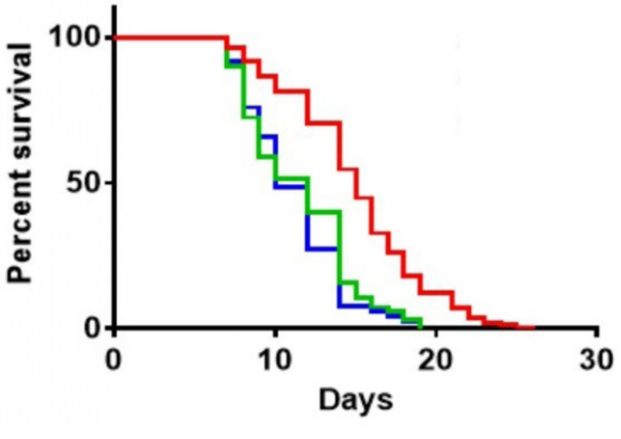

Figure S1: The *C. elegans* strain GRU102 constitutively expresses full-length human Aβ1–42, resulting in a notably decreased lifespan and diminished health-span. Kaplan-Meier survival analysis was conducted on Aβ-expressing nematodes at different temperatures, revealing that GRU102 strain nematodes exhibited a significantly reduced lifespan at 25°C compared to those maintained at 22.5°C, while nematodes at 20°C displayed a normal lifespan. This difference was statistically significant (log-rank test: $p<0.001$). Note: (—)-22.5°C; (—)-20°C; (—)-25°C.

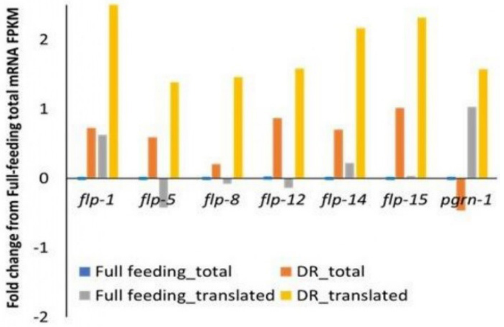

Figure S2: Total versus translated mRNA for progranulin and neurotransmitters under dietary restriction conditions: RNAseq data analysis identifies genes that are translationally regulated under DR. For *pgrn-1* gene under DR results show that translation is upregulated and for flp genes both transcription and translation are increased. Fold change in total and translated (polysome-associated) transcript levels compared to full fed control total mRNA levels for the genes shown. All RNAseq experiments were carried out in four biological replicates. Control values used to zero average results. Test is DR animals; control is fully fed. FPKM=Fragments per Kilobase Megabase. Note: (■)-Full feeding_total; (■)-DR_total; (■)-Full feeding_translated; (■)-DR_translated.

Correspondence to: Dilawar Ahmad Mir, Kathryn W. Davis Center for Regenerative Biology and Aging, Mount Desert Island Biological Laboratory, Maine, United States of America, E-mail: dilawerjk@gmail.com

Received: 27-Apr-2024, Manuscript No. JCMS-24-25577; **Editor assigned:** 30-Apr-2024, Pre QC No. JCMS-24-25577 (PQ); **Reviewed:** 14-May-2024, QC No. JCMS-24-25577; **Revised:** 21-May-2024, Manuscript No. JCMS-24-25577 (R); **Published:** 28-May-2024, DOI: 10.35248/2593-9947.24.8.276

Citation: Mir DA, Cox M, Horrocks J, Ma Z, Rogers A (2024) Roles of Progranulin and FRamides in neural *versus* non-neural tissues on Dietary Restriction-Related Longevity and Proteostasis in *C. elegans*. J Clin Med Sci. 8:276.

Copyright: © 2024 Mir DA, et al. This is an open-access article distributed under the terms of the Creative Commons Attribution License, which permits unrestricted use, distribution, and reproduction in any medium, provided the original author and source are credited.

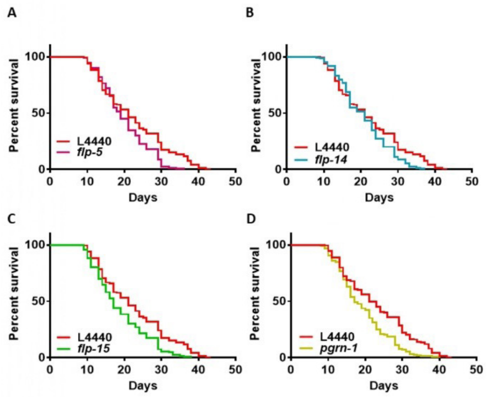

Figure S3: This figure depicts the outcomes of RNAi targeting the *pgrn-1* and *flp* genes in the DA465 strain (eat-2 mutant) to evaluate their effects on lifespan. The bar graphs demonstrate a detrimental effect on the lifespan of eat-2 mutants when subjected to RNAi targeting the *pgrn-1* and *flp* genes. Compared to the DR control, knockdown of *pgrn-1* and *flp* genes resulted in decreased lifespan. The percentage survival of minimum lifespan and associated P-values from the log-rank test, comparing with the controls, are as follows. Note: (A) *pgrn-1*(-19.04%, n=151); (B) *flp-5* (-9.52%, n=153); (C) *flp-14* (0%, n=124); (F) *flp-15* (-19.04%, n=148). Kaplan-Meier survival curves were compared using the Mantel-Cox log-rank test.

Table S1: List of strains that were used in this study.

Strain name	Genotype	Backcrossed	Source
N2	Wild type	n/a	CGC
TU3335	lin-15B(n744) X; uIs57	n/a	CGC
DA465	eat-2(ad465) II	n/a	CGC
GRU102	gnaIs2 [myo-2p::YFP + unc-119p::Abeta1-42]	n/a	CGC
ANR201	lin-15B(n744) X; uIs57; gnaIs2 [myo-2p::YFP + unc-119p::Abeta1-42].	3×	This study

Table S2: Survival of the TU3335 under AL vs. DR.

RNAi	Rep	AL/DR	N-value	Median lifespan	% Survival of Median Lifespan Control vs. RNAi under AL vs. AL and DR vs. DR	Mantel-Cox test (P value) of Control vs. RNAi under DR vs. DR and AL vs. AL	% Survival of Median Lifespan (AL vs. DR)	Mantel-Cox test (P value) (AL vs. DR)	Max. Lifespan
ctl	A	AL	111	14			28.57	< 0.0001	23
		DR	172	18					27
	B	AL	113	14			25	< 0.0001	24
		DR	156	17.5					25
	C	AL	111	11			36.36	< 0.0001	18
		DR	172	15					23
	A	AL	152	15	7.14	< 0.0001	20	< 0.0001	23
		DR	175	18	0	ns			28
flp-5	B	AL	118	15	7.14	ns	13.33	< 0.0001	22
		DR	148	17	-2.85	ns			30
	C	AL	152	12	9.09	0.0022	33.33	< 0.0001	24
		DR	175	16	6.66	< 0.0001			25
	A	AL	164	15	7.14	0.001	20	< 0.0001	22
		DR	168	18	0	ns			27

	B	AL	119	15	7.14	<0.0001	20	< 0.0001	19
		DR	175	18	2.85	ns			27
flp-14	C	AL	186	12	6.09	0.0002	75	< 0.0001	22
		DR	169	21	40	<0.0001			30
	A	AL	149	14	0	ns	28.57	< 0.0001	22
		DR	141	18	0	ns			27
	B	AL	136	15	7.14	ns	20	< 0.0001	19
		DR	153	18	2.85	0.0226			28
flp-15	C	AL	131	13	18.18	< 0.0001	46.1	< 0.0001	21
		DR	157	19	26.66	< 0.0001			30
	A	AL	186	14	0	ns	28.57	< 0.0001	21
		DR	169	18	0	ns			28
pgrn-1	B	AL	101	14	0	ns	28.57	< 0.0001	19
		DR	127	18	2.85	ns			28
	C	AL	107	14	27.27	< 0.0001	42.85	< 0.0001	21
		DR	139	20	33.33	< 0.0001			

Table S3: Survival of the N2 Wild type under AL vs. DR

RNAi	Rep	AL/DR	N-value	Median lifespan	% Survival of Median Lifespan Control vs. RNAi under AL vs. AL and DR vs. DR	Mantel-Cox test (P value) of Control vs RNAi under DR vs. DR and AL vs. AL	% Survival of Median Lifespan (AL vs. DR)	Mantel-Cox test (P value) (AL vs. DR)	Max. Lifespan
ctl	A	AL	166	15			46.66	<0.0001	26
		DR	97	22					37
	B	AL	71	17			17.64	0.0133	31
		DR	65	20					40
flp-5	A	AL	206	16	0.66	0.0011	37.27	<0.0001	29
		DR	99	22	0	ns			38
	B	AL	62	18	5.88	ns	22.22	ns	37
		DR	68	22	10	0.0158			28
flp-14	A	AL	159	15	0	0.0096	20	ns	30
		DR	111	18	-18.18	<0.0011			27
	B	AL	88	14	-17.64	0.0023	39.2	<0.0001	29
		DR	90	19.5	-2.5	0.0467			32
flp-15	A	AL	180	16	6.66	0.0028	27.27	<0.0001	23
		DR	122	22	0	ns			39
	B	AL	88	17	0	ns	29.41	0.0019	35
		DR	81	22	10	ns			36
pgrn-1	A	AL	179	15	0	ns	26.66	<0.0001	27
		DR	137	19	-13.63	<0.0001			36
	B	AL	78	17	0	ns	23.52	0.0008	34
		DR	74	21	5	ns			35

Table S4: Survival of the eat-2 mutant.

RNAi	N-val	Median lifespan	Max. Lifespan	% Survival of Median Lifespan	Mantel-Cox test (P value)
ctl	138	21	43		0.0003
flp-5	153	19	36	-9.52	
flp-14	124	21	37	0	0.0106
flp-15	148	17	38	-19.04	<0.0001
pgrn-1	151	17	40	-19.04	<0.0001

Table S5: eat-2 Survival statistics of the heat stress recovery assay.

Strain eat-2 on RNAi	Rep	N-value	% Survival after 3 days of Heated @35 C	Average % of Survival	% of Heat Resistance	t-test Heated (Ctl vs heated RNAi)
Ctl	A	120	76.90%	85.10%		
	B	112	90.20%			
	C	119	88.20%			
flp-5	A	115	91.30%	86.00%	0.90%	ns
	B	113	84.10%			
	C	86	82.60%			
flp-14	A	96	92.70%	89.47%	4.37%	ns
	B	117	88.00%			
	C	114	87.70%			
flp-15	A	110	85.50%	85.07%	-0.03%	ns
	B	113	85.80%			
	C	118	83.90%			
pgrn-1	A	103	86.40%	76.83%	8.27%	ns
	B	125	84.80%			
	C	108	59.30%			
